# Permissive Weight Bearing in Proximal Humeral Fracture Management: A Survey-Based Inquiry in the Netherlands

**DOI:** 10.7759/cureus.57670

**Published:** 2024-04-05

**Authors:** Amber Hameleers, Bert Boonen, Jasper Most, Martijn Dremmen, Martijn G.M. Schotanus, Raoul Van Vugt

**Affiliations:** 1 Department of Orthopedic Surgery, Zuyderland Medical Center, Sittard-Geleen, NLD; 2 Department of Surgery, Maastricht University Medical Center+, Maastricht, NLD; 3 Department of Rehabilitation Medicine, Zuyderland Medical Center, Heerlen, NLD; 4 Department of Surgery, Zuyderland Medical Center, Heerlen, NLD

**Keywords:** trauma surgery, treatment, survey, permissive weight bearing, proximal humerus fractures

## Abstract

Purpose

Proximal humeral fractures (PHF) are common, particularly among the elderly due to low-energy trauma. Adequate rehabilitation is essential for functional recovery, whether through conservative or surgical treatment. Permissive weight bearing (PWB) is a relatively new rehabilitation concept, characterized by earlier mobilization of the affected limb/joint after trauma. Multiple studies demonstrated the value of PWB for the lower extremities, but this has not been translated to the upper extremity (i.e. PHF). Therefore, our aim was to investigate the current state and variability of rehabilitation of PHF and the role of implementing PWB principles in aftercare.

Materials and methods

An online survey, comprising 23 questions about the treatment of PHF, was distributed amongst an estimated 800 Dutch orthopaedic and trauma surgeons via the Dutch Orthopaedic and Dutch Trauma Society newsletter from May 2021 until July 2021.

Results

Among 88 respondents (n=69 orthopaedic, n=17 trauma surgeons, and n=2 other), most recommended early post-trauma mobilization (<6 weeks). Additionally, 53.4% (n=49) advised starting load bearing after six weeks for conservatively treated patients and 59.8% (n=52) for operative treatment. A wide variation of exercises used after immobilization was found in both groups. The usage of a sling after operative treatment was advised by 86% (n=74) of all 86 respondents.

Conclusions

The present study found limited consensus about PHF aftercare and the implementation of weight-bearing principles. The majority recommended early mobilization and advised the usage of a sling. A protocol capable of accommodating the diversity in aftercare (e.g. fracture type) is essential for maintaining structured rehabilitation, with PWB emerging as a promising example. More prospective studies are needed to form an evidence-based protocol focusing on the aftercare of PHF.

## Introduction

Proximal humeral fractures (PHF) are common and are mostly caused by low-energy injuries in the elderly population. It is the third most prevalent non-vertebral fracture, and 10-20% of these fractures require operative treatment [1,2]. In the Western world, PHF are estimated to account for 6% of all fracture types [3]. An older age is associated with more complex fracture types, which are often caused by ground-level falls on an outstretched arm. Moreover, the elderly population is more susceptible to prolonged immobilization during treatment causing suboptimal rehabilitation in the overall function of the shoulder joint [3,4]. As a result of poor treatment, PHF could lead to loss of function of the shoulder joint and chronic pain symptoms.

To regain the best function possible, a good rehabilitation strategy is essential, regardless of whether it is treated conservatively or operatively [5]. Permissive weight bearing (PWB) is a relatively new rehabilitation concept, characterized by earlier mobilization of the affected limb/joint after trauma where the course of treatment is most dependent on the patient's ability to allow weight bearing and the instructions given by the physiotherapist. For example, the ability to put weight on the joint depends on swelling, pain, and the feeling of instability of the joint [6,7]. This implies that not time itself is the primary factor in determining the loading capacity of the limb, but mainly patient factors guide the capacity to do so, leading to a more individualized, non-time congruent, rehabilitation approach. PWB is defined as the next step after a period of immobilization, namely, by including additional (body) weight into movement (carrying, getting up from a chair, etc.). Despite the fact that there are multiple studies investigating the effect of PWB on the lower extremities [7-9], there is a lack of evidence regarding PWB for the upper extremities.

The purpose of this present national survey was to investigate the current state and variability of rehabilitation treatment regimens for patients with PHF amongst Dutch orthopaedic and trauma surgeons. The survey aims to determine which protocols and criteria are used for rehabilitation treatment, specifically including PWB if used.

## Materials and methods

The authors have developed a comprehensive survey aimed at gathering more insights about the treatment of proximal humeral fractures from orthopaedic and trauma surgeons in the Netherlands. The survey comprised 23 questions, systematically divided into three sections, each designed to explore diverse facets of PHF treatment. The inclusion criteria were all orthopaedic surgeons and general surgeons with a specialization in trauma (trauma surgeons) who operate proximal humeral fractures in the Netherlands. The exclusion criteria were students and surgeons in training, while they have less (long-term) experience with PHF.

The initial section of the survey focused on the professional discipline and expertise of surgeons, particularly emphasizing the decision-making processes regarding conservative or surgical treatment modalities. Additionally, the survey aimed to determine the impact of established guidelines, including the Arbeitsgemeinschaft für Osteosynthesefragen (AO) guidelines (Section 6.2.1), Hertel classification, and Neer classification [5,10,11], on the decision-making process in clinical settings.

The subsequent sections were dedicated to the examination of two primary treatment approaches: conservative and operative treatment with specific reference to the integration of PWB in the rehabilitation period. 

After permission was obtained from the boards of the Dutch Trauma Society (NVT) and the Dutch Orthopedic Society (NOV), the online questionnaire was distributed in May 2021 among Dutch orthopaedic and trauma surgeons via the newsletter of the NVT and the NOV. A total of 750 orthopaedic and 145 trauma surgeons work in the Netherlands, the majority of whom are affiliated with the NVT and/or NOV. The survey targeted the entire cohort of orthopaedic and trauma surgeons who had experience with PHF. The survey was online from May 2021 until August 2021. Unfortunately, it was not possible to send a reminder while the survey was still online.

Statistical analysis

All analyses were performed with Statistical Product and Service Solutions (SPSS, version 29.0; IBM Corp., Armonk, NY) software. Descriptive statistics were used to display the demographic data, and the results were displayed as frequencies with percentages for categorical variables.

## Results

Physicians' characteristics are shown in Table 1. Of the 88 surgeons who responded (estimated response rate of 10%), 69 (79.3%) were orthopaedic surgeons, 17 (19.5%) were trauma surgeons, and one filled in other, which was not further defined (Appendix, Q1). Most of the respondents (48.9%) have been working in the field for more than 10 years (Q2).

**Table 1:** Physicians' characteristics n = number of respondents, PHF = proximal humeral fractures

Total respondents, n (%)	88 (100%)
Discipline, n (%)	
Orthopaedic surgeon	69 (79.3)
Trauma surgeon	17 (19.5)
Other	1 (1.1)
Number of years working, n (%)	
< than 5 years	29 (33)
Between 5 and 10 years	16 (18.2)
More than 10 years	43 (48.9)
Number of PHF operations annually, n (%)	
<5 operations	33 (37.5)
Between 5 and 10 operations	23 (26.1)
Between 10 and 25 operations	26 (29.5)
> 25 operations	5 (5.7)
Use of classification systems, n (%)	
AO guidelines	22 (25.2)
Hertel classification	7 (8.0)
Neer classification	30 (34.1)
No classification system	26 (29.9)
Other	2 (2.3)
Main indication to operate, n (%)	
Patient factors (age/demands)	39 (44.3)
Dislocation of displacement	48 (54.5)
Amount of fractured parts	1 (1.1)
Most frequently reported trauma mechanism, n (%)	
Low-energy trauma	86 (98.9)
Value of sling usage in the healing process, n (%)	
Yes, I advise the patient to wear a sling	74 (86.0)

Conservative treatment

To decide when to start with mobilization in conservative treatment, the vast majority base their choice on clinical experience (44.3%) or local protocols used in the respective hospital (31.8%), while only 8% of the respondents use the AO guidelines (5) (Q10). The duration of immobilization is mostly determined by pain complaints (54.5%) and fracture type (30.7%) (Q9). For conservatively treated PHF, 35.2% of the surgeons advise immobilization for less than two weeks, 30.7% advise immobilization between two and three weeks, 29.5% between four and six weeks, 3.4% recommend six weeks or longer, and three surgeons (3%) suggested no immobilization at all (Q8). By self-report, 25 (34.1%) of all respondents saw less than 1% complications, 53 (44.3%) between 1% and 5%, six (14.8%) between 5% and 10%, and one (3.4%) > 10% complications after starting earlier mobilization (<6 weeks) (Q14).

**Figure 1:**
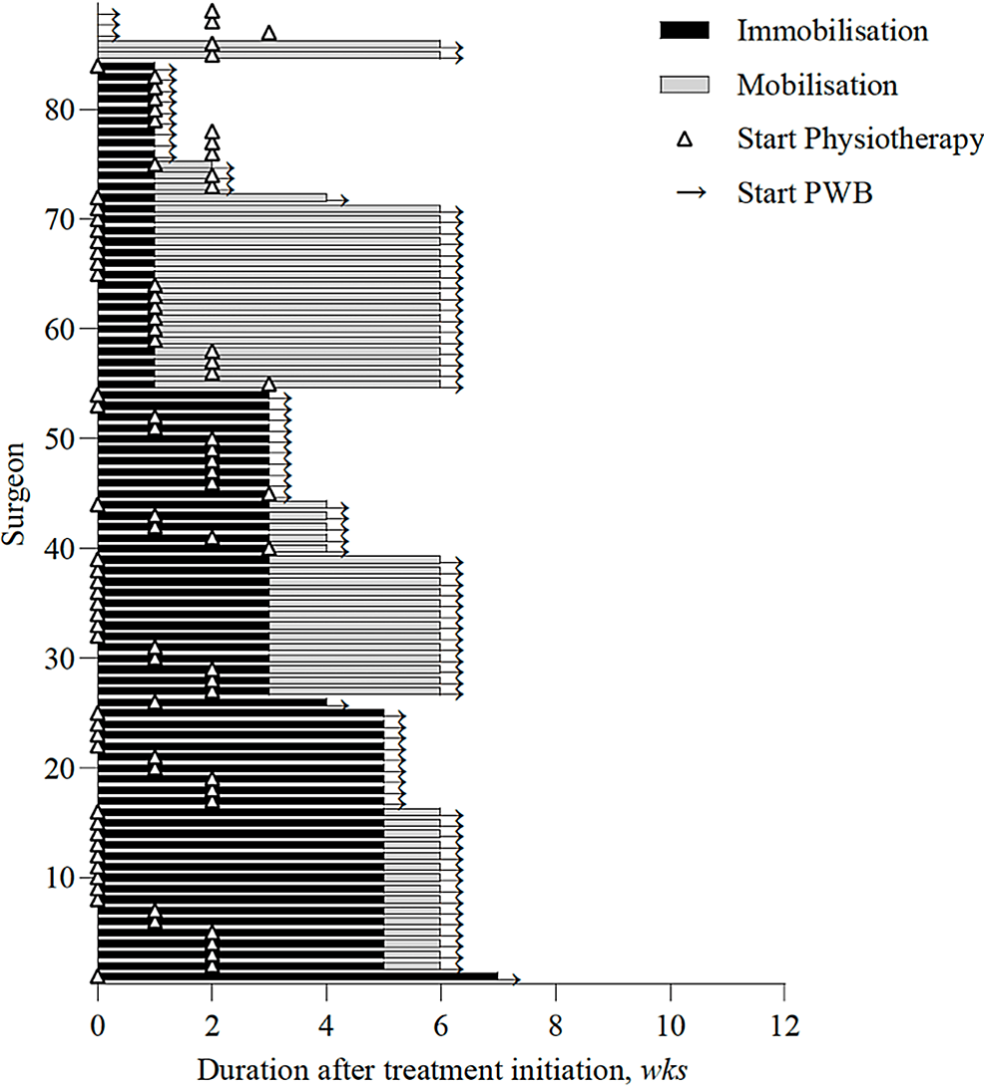
Variation in treatment options and duration of the initiation of movement in weeks PWB = permissive weight bearing

Surgeons seem to be inconsistent in the timing of referral to a physiotherapist; 31.8% recommend consultation immediately after trauma, 37.5% after a period of immobilization, almost 26.1% would base referral on patient factors, and 4.5% would not refer (Q11). The surgeons who recommended physiotherapy considered starting with pendulum exercises or gradually progressive training of the affected arm (Figure 1) (Q12).

Most surgeons instructed their patients to start load-bearing exercises six weeks after trauma (53.4%), and 27.2% would decide at what time load-bearing exercises were applicable based on fracture type (Q13).

Operative treatment

For operatively treated patients, the period of immobilization was based on local protocol according to 43.2% of the surgeons, while 37.5% based their choice on clinical experience. Similar to the conservatively treated patient, 7% of the respondents adhere to the AO guidelines (5) (Q18). The majority (43.7%) indicated that the most important reason for immobilization was the stability of the osteosynthesis material (OSM). Other reasons were pain complaints (26.4%), type of fracture (20.7%), or protocol (8.0%) (Q17).

Mobilization occurred earlier (after surgery) than for conservatively treated patients (after trauma). The data indicate that 28.7% advised immobilization for less than one week after surgery, 34.5% between one and two weeks, 19.5% between two and four weeks, and 17.2% between four to six weeks (Q16).

Thirty (28.4%) respondents indicated less than 1% complications after starting early mobilization, 39 (60.2 %) between 1% and 5%, 13 (6.8%) between 5% and 10%, and three (1.1%) in more than 10% of the cases (Q22).

Additionally, 56.8% of the surgeons would send their patients directly after surgery to physiotherapy training (Q19). A wide variation in the usage of different types of exercises (non-load bearing and load bearing) used after immobilization was found (Q20). Moreover, 52 surgeons (59.8%) recommended starting load-bearing exercises after six weeks compared to 20 (22.7%) surgeons who would let the start of load-bearing exercises depend on fracture type (Q21). The functional outcome of patients with a PHF is mostly determined based on physical examination/performance testing (56.8%) according to the physicians (Q23).

## Discussion

Main findings

The present survey was designed to assess the current state of treatment for conservatively and operatively treated PHF in the Netherlands. The results show a wide variety of post-operative and conservative treatment options. The present study found that, for both conservative and operative treatments, surgeons deviate from established guidelines. The majority of the respondents advocate for early mobilization, starting after a period of less than two weeks of immobilization for conservatively treated patients and around one week for those undergoing operative treatment. Load bearing is typically initiated six weeks after trauma in both groups, with a strong recommendation for sling usage during this period. Furthermore, decisions regarding the choice of exercise and timing of start differed immensely.

Interpretation of the results

In our study group, we observed that surgeons often deviate from established guidelines and utilize different ones when it comes to the treatment of PHF. It is often assumed that surgeons use the AO guidelines or the Neer classification to provide the best possible care for their patients [12-14]. Almost 35% of the respondents use the Neer classification, while 25.2% adhere to the AO guidelines. While the AO guidelines focus on the vascular supply of the humeral head and its prognostic factors for possible avascular osteonecrosis, Neer’s system reviews the number of fracture parts and displacement from each other [5]. The third well-known classification system, the Hertel classification, is based on describing the fracture using five different fracture planes [10,15]. Although a significant portion of the respondents opted for a classification system as the basis for their treatment, almost 30% did not use one. This could be due to the fact that the variety of fracture patterns makes it challenging to fit every case into predefined categories.

Additionally, no consensus was observed between the conservative and operative treatment groups concerning various aftercare aspects. The difference between the two groups with regard to the duration of immobilization can be explained by several factors (e.g. the stability of the fracture). In the operative group, the fracture is stabilized using osteosynthesis materials, making the fracture almost immediately stable, providing the possibility to start mobilization at an earlier stage [16,17]. In the conservative group, excessive movement in the early stages of fracture healing can lead to fracture dislocation. This indeed is reflected in the present survey, where surgeons indicate earlier mobilization after surgery as compared to conservative treatment. Secondly, pain (54.5%) does seem to play a more significant role in the duration of immobilization in the conservative group, unlike the operative group where it is based on local protocol (44.2%) or experience (38%). As mentioned before, the operative approach provides a more stabilized environment for the healing process, allowing the postoperative course to focus more on the surgical techniques used and the duration required for bone healing. One might question whether the use of analgesics after surgery could impact the aforementioned outcomes for conservatively treated patients as they often will not receive the same painkillers.

Furthermore, variations are evident in both the timing of referrals to physical therapists and the array of exercises applied during the rehabilitation process. Most of the surgeons in both groups would send their patients to a physiotherapist after trauma. The timing of referral differed mainly in the conservative group, where one-third would refer their patients directly after trauma and one-third would wait until after a period of immobilization. The variation can be explained by the fact that the conservative group more often consists of patients with less severe fractures and thus not also needing guidance during rehabilitation.

In daily practice, it is often the physiotherapist who guides the patients through the exercises and adjusts the treatment plan based on the patient's progress and needs. We observe the same trend when examining the initiation of load bearing. Although half of the respondents opt for adhering to a protocol, a significant portion chooses for either faster mobilization or bases their decision on the fracture type, thus focusing more on patient characteristics. More than half of the surgeons advise starting load-bearing exercises six weeks after trauma/operative treatment, which is in line with the AO guidelines [5]. In the past, it was thought that early mobilization could lead to complications such as fracture displacement. Over the last couple of decades, PWB has shown promising results in lower extremity fracture recovery [7,9,18,19]. However, this promising trend has not been translated to comparable trauma in the upper extremity nor for PHF. Earlier (partial) load bearing of the lower extremities could allow patients to return to work sooner and prevent them from having mental health issues due to immobilization and not being able to engage in society [20,21]. Other than allowing patients to return to work faster, early mobilization also does not seem to cause more complications, which is in line with our findings [22]. The main purpose of the survey was to give an overview of the treatment options and variability associated with it in the Netherlands and should not be seen as a guideline for preferred methodology.

Limitations

This study has a couple of important limitations. Firstly, our response rate was approximately 10%. Secondly, we did not focus on the different ranges of experiences, patient characteristics (e.g. fracture type), or the differences between answers given by trauma versus orthopaedic surgeons. Thirdly, the complication rates discussed estimated numbers based on the surgeon’s experience after treating patients with such fractures. It should be kept in mind that the surgeon's experience with complications was not objectified and therefore might deviate from actual practice.

## Conclusions

The present survey showed that there is limited consensus about aftercare and the role of load bearing after trauma regarding PHF. Load bearing usually starts six weeks after trauma, and early mobilization does not seem to cause more complications. The absence of consensus prevails within the medical community concerning the precise terminology and definition of PWB. The unavailability of a standardized interpretation leads to inconsistency in clinical application, particularly in upper extremity injuries, such as PHF. PWB or load bearing has the potential to foster early mobilization and functional recovery. The diversity in fracture types, osteosynthesis materials, differences in terminology, and patient characteristics necessitates a protocol that accommodates such variability while providing guidelines for maintaining structured rehabilitation. Permissive weight or load bearing stands out as a promising example in this regard.

## Appendices

Appendix 1: The survey 

First Section: General Information

1. What is your discipline?

a. Orthopaedic surgeon

b. Trauma surgeon

c. Other

2. How long have you been a surgeon?

a. Less than 5 years

b. Between 5 and 10 years

c. More than 10 years

3. How often do you operate a proximal humeral fracture on a yearly basis?

a. Less than 5 times per year

b. Between 5 and 10 times per year

c. Between 10 and 25 times per year

d. More than 25 times per year

4. Do you use a classification system for proximal humeral fractures? If so, which one do you use?

a. I don’t use a specific type of classification

b. Yes, I use the AO guidelines

c. Yes, I use the Neer classification

d. Yes, I use the Hertel classification

e. Other

5. What is the main indication to operate a humeral fracture?

a. Dislocation or displacement

b. Amount of fracture parts

c. Patient factors (age/demands)

6. What is in most cases the mechanism of injury?

a. Low-energy trauma

b. High-energy trauma

7. The usage of a shoulder sling adds significant value to the further healing process directly after operative treatment of a proximal humeral fracture.

a. Agree, I advise patients to wear a sling after operative treatment

b. Disagree, I barely advise patients to wear a shoulder sling after operative treatment

Second Section: Proximal Humeral Fractures Treated Conservatively

8. Duration of shoulder immobilization of patients with proximal humeral fractures:

a. Immediately

b. Less than two weeks

c. Between 2 and 4 weeks

d. Between 4 and 6 weeks

e. 6 weeks or longer

9. The duration of immobilization depends mostly on:

a. Type of fracture

b. Patient age

c. Callus formation on x-ray

d. Pain complaints

e. Protocol

f. Other

10. When starting with mobilization, which guideline do you follow?

a. Local protocol

b. AO guidelines

c. Clinical experience

d. As learned from trainer

e. Gut feeling

11. When do you refer the patient to a physiotherapist?

a. I do not refer

b. Immediately

c. After a period of immobilization

d. Depending on patient factors

12. Which exercises do you advise patients to start with after immobilization of a proximal humeral fracture?

a. Passive range of motion exercises

b. Assisted motion exercises

c. Pendulum exercises

d. Isometric exercises

e. Gradually progressive functional use of the arm

f. All of the above

g. Other

13. When do you start with load/weight-bearing exercises after a proximal humeral fracture?

a. Immediately

b. After 2 weeks

c. After 4 weeks

d. After 6 weeks

e. Depending on fracture type

f. Physiotherapist decides

14. How often do you see complications related to overload of the shoulder joint, such as infection, migration of fracture parts or dislocation/displacement, due to early mobilization?

a. <1 %

b. Between 1-5%

c. Between 5-10%

d. More than 10%

15. How do you determine the (functional) outcome of patients with proximal humeral fractures that are treated conservatively?

a. Based on anamnestic information

b. Based on physical examination (performance testing)

c. Based on the information the physiotherapist has given about the patient

d. Based on imaging techniques (X-ray, MRI, CT)

Third Section: Proximal Humeral Fractures Treated Operatively (Only Osteosynthesis)

16. Duration of shoulder immobilization of patients with proximal humeral fractures:

a. Less than a week

b. Between 1 and 2 weeks

c. Between 2 and 4 weeks

d. Between 4 and 6 weeks

e. 6 weeks or longer

17. The duration of immobilization depends mostly on:

a. Type of fracture

b. Patient age

c. Callus formation on X-ray

d. Pain complaints

e. Stability of OSM

f. Protocol

18. When starting with mobilization, which guideline do you follow?

a. Local protocol

b. AO guidelines

c. Clinical experience

d. As learned from trainer

e. Gut feeling

19. When do you refer the patient to the physiotherapist?

a. I do not refer

b. Immediately post-op

c. After a period of immobilization

d. Depending on patient factors

20. Which exercises do you advise patients to start with after immobilization of a proximal humeral fracture?

a. Passive range of motion exercises

b. Pendulum exercises

c. Isometric exercises

d. Gradually progressive functional use of the arms

e. All of the above

f. Other

21. When do you start with weight-bearing exercises after a proximal humeral fracture?

a. Immediately

b. After 2 weeks

c. After 4 weeks

d. After 6 weeks

e. Depending on fracture type

f. Physiotherapist decides

22. How often do you see complications related to early mobilization?

a. <1 %

b. Between 1-5%

c. Between 5-10%

d. More than 10%

23. How do you determine the (functional) outcome of patients with a proximal humeral fracture who are treated operatively?

a. Based on anamnestic information

b. Based on physical examination (performance testing)

c. Based on the information the physiotherapist has given about the patient

d. Based on imaging techniques (X-ray, MRI, CT)
